# Cost-effectiveness of a multitarget stool DNA test for colorectal cancer screening of Medicare beneficiaries

**DOI:** 10.1371/journal.pone.0220234

**Published:** 2019-09-04

**Authors:** Steffie K. Naber, Amy B. Knudsen, Ann G. Zauber, Carolyn M. Rutter, Sara E. Fischer, Chester J. Pabiniak, Brittany Soto, Karen M. Kuntz, Iris Lansdorp-Vogelaar

**Affiliations:** 1 Erasmus MC, University Medical Center Rotterdam, Department of Public Health, Rotterdam, The Netherlands; 2 Institute for Technology Assessment, Massachusetts General Hospital, Boston, Massachusetts, United States of America; 3 Department of Epidemiology and Biostatistics, Memorial Sloan Kettering Cancer Center, New York, New York, United States of America; 4 RAND Corporation, Santa Monica, California, United States of America; 5 Kaiser Permanente Washington Health Research Institute, Seattle, Washington, United States of America; 6 Division of Health Policy and Management, School of Public Health, University of Minnesota, Minneapolis, Minnesota, United States of America; Beckman Research Institute, UNITED STATES

## Abstract

**Background:**

In 2014, the Centers for Medicare and Medicaid Services (CMS) began covering a multitarget stool DNA (mtSDNA) test for colorectal cancer (CRC) screening of Medicare beneficiaries. In this study, we evaluated whether mtSDNA testing is a cost-effective alternative to other CRC screening strategies reimbursed by CMS, and if not, under what conditions it could be.

**Methods:**

We use three independently-developed microsimulation models to simulate a cohort of previously unscreened US 65-year-olds who are screened with triennial mtSDNA testing, or one of six other reimbursed screening strategies. Main outcome measures are discounted life-years gained (LYG) and lifetime costs (CMS perspective), threshold reimbursement rates, and threshold adherence rates. Outcomes are expressed as the median and range across models.

**Results:**

Compared to no screening, triennial mtSDNA screening resulted in 82 (range: 79–88) LYG per 1,000 simulated individuals. This was more than for five-yearly sigmoidoscopy (80 (range: 71–89) LYG), but fewer than for every other simulated strategy. At its 2017 reimbursement rate of $512, mtSDNA was the most costly strategy, and even if adherence were 30% higher than with other strategies, it would not be a cost-effective alternative. At a substantially reduced reimbursement rate ($6–18), two models found that triennial mtSDNA testing was an efficient and potentially cost-effective screening option.

**Conclusions:**

Compared to no screening, triennial mtSDNA screening reduces CRC incidence and mortality at acceptable costs. However, compared to nearly all other CRC screening strategies reimbursed by CMS it is less effective and considerably more costly, making it an inefficient screening option.

## Introduction

Colorectal cancer (CRC) is the second most common cause of cancer death in the United States [1]. Randomized trials of fecal occult blood tests (FOBTs) and flexible sigmoidoscopy have shown that screening can effectively reduce both CRC incidence [2–6] and mortality [2–12]. In June 2016, the US Preventive Services Task Force updated their CRC screening recommendations, including guidelines on the use of FOBTs, flexible sigmoidoscopy, colonoscopy, and the recently-developed multitarget stool DNA test (mtSDNA) [13].

In April 2014, Imperiale et al. [14] published the findings of a study evaluating the test performance of the mtSDNA test, Cologuard (Exact Sciences Corporation), based on a single round of screening. Cologuard combines DNA assays for multiple aberrant gene mutations and a proprietary fecal immunochemical assay. Compared with a fecal immunochemical test (FIT), Cologuard demonstrated higher sensitivity for CRC and advanced adenomas but lower specificity. In October 2014, the Centers for Medicare and Medicaid Services (CMS) granted coverage of Cologuard once every 3 years (the interval recommended by the manufacturer) for asymptomatic, average-risk Medicare beneficiaries [15]. Following CMS’s Clinical Laboratory Fee Schedule (CLFS), reimbursement for Cologuard was set by “crosswalking” to comparable diagnostic tests already on the CLFS. This process yielded a 2014 reimbursement rate for Cologuard of $492.72 per test [16].

CMS requested an analysis of mtSDNA screening of Medicare enrollees from the MITRE Corporation. MITRE commissioned investigators from the Cancer Intervention and Surveillance Modeling Network (CISNET) to assess whether mtSDNA testing is a cost-effective alternative to other CRC screening strategies available to Medicare beneficiaries, and if not, to assess at what reimbursement rate, level of screening uptake, or screening interval it could be a cost-effective option.

## Methods

### CISNET models

We used three independently-developed microsimulation models of CRC from the National Cancer Institute’s CISNET consortium—the CRC Simulated Population Model for Incidence and Natural History (CRC-SPIN), Microsimulation Screening Analysis for CRC (MISCAN), and Simulation Model of CRC (SimCRC)—to evaluate the cost-effectiveness of screening Medicare beneficiaries with the mtSDNA test, or with an alternative CRC screening approach. All models describe the natural history of CRC in an unscreened population, based on the adenoma-carcinoma sequence [17–19]. Simulated persons enter free of colonic and rectal lesions at age 20. As they age, they are at risk of developing adenomas. Each adenoma may grow in size, and some may transition to a preclinical (i.e., undiagnosed) CRC. Preclinical cancers may become symptomatic, at which point the person becomes a clinically-detected case. Persons may die from causes other than CRC at any age, and persons with detected CRC may die from the disease.

Each model has a screening component that allows the natural history of CRC to be interrupted due to the detection of a preclinical cancer or the detection and removal of adenoma(s). The chance that screening detects asymptomatic disease in a simulated person depends on the sensitivity of the screening test and, for endoscopic tests, whether the lesion is within the reach of the scope. Screened persons without an underlying lesion may have a false-positive test result and undergo an unnecessary follow-up colonoscopy. Non-adenomatous polyps (e.g., hyperplastic polyps) are not modeled explicitly, but their detection is reflected in the false-positive rates of the tests. The impact of screening depends on the characteristics of the test performed, and on how frequently it is repeated.

### CRC screening

We used the models to simulate cohorts of previously unscreened US 65-year-olds, with mtSDNA every 3 years (as specified in the final coverage determination) and six other strategies available to Medicare beneficiaries [20]: annual fecal occult blood testing (FOBT) with either a high-sensitivity guaiac-based FOBT (gFOBT) or a FIT, 5-yearly flexible sigmoidoscopy, 10-yearly flexible sigmoidoscopy with annual gFOBT or annual FIT, and 10-yearly colonoscopy. We assumed all screening begins at age 65 and ends no later than age 75. Individuals with a positive non-colonoscopy screening test undergo a follow-up colonoscopy. Individuals with adenomas detected at a screening or follow-up colonoscopy transition to a surveillance regimen [21], with colonoscopy performed every 3 or 5 years (dependent on findings) until at least age 85.

For the base-case analysis, we assumed 100% adherence to all screening, follow-up, and surveillance procedures to assess the effect of screening in those willing to be screened. Alternative assumptions were explored in sensitivity analyses.

Assumptions about test accuracy were based on published findings (Table 1). Complication risks from colonoscopy were obtained from a study by Van Hees et al. [22] that estimated excess risks of serious gastrointestinal events, other gastrointestinal events, and cardiovascular events by age and polypectomy status among Medicare beneficiaries undergoing colonoscopy compared with a matched control group that did not have colonoscopy [23].

**Table 1:** Test characteristics* used in the analysis.

			Sensitivity Analysis		
Screening Test Test Characteristic	Base-Case Value, %	Source	Worst-Case Value, %	Best-Case Value, %	Source
mtSDNA		Imperiale et al, 2014[14]			Imperiale et al, 2014[14]
Specificity†	89.8		Not varied	Not varied	Not varied
Sensitivity for adenomas <10 mm	17.2‡		15.9‡	18.6‡	
Sensitivity for adenomas ≥10 mm	42.4§		38.7§	46.2§	
Sensitivity for colorectal cancer	92.3		84	97	
FIT (cutoff 20 μg of hemoglobin per g of feces)		Imperiale et al, 2014[14]			Imperiale et al, 2014[14]
Specificity†	96.4		Not varied	Not varied	Not varied
Sensitivity for adenomas <10 mm	7.6‡		6.7‡	8.6‡	
Sensitivity for adenomas ≥10 mm	23.8§		20.8§	27§	
Sensitivity for colorectal cancer	73.8		62.3	83.3	
gFOBT		Zauber et al, 2008[24]			
Specificity†	92.5		Not varied	Not varied	Not varied
Sensitivity for adenomas <6 mm	7.5‖		7.5‖	7.5‖	Zauber et al, 2008[24]
Sensitivity for adenomas 6–9 mm	12.4		10	26.2	Zauber et al, 2008[24]
Sensitivity for adenomas ≥10 mm	23.9		17.7	49.4	Zauber et al, 2008[24]
Sensitivity for colorectal cancer	70		61.5	79.4	Levi et al, 2011[25] Allison et al, 1996[26]
Colonoscopy (within reach)¶					
Specificity†	86**	Schroy et al, 2013[27]	Not varied	Not varied	Not varied
Sensitivity for adenomas <6 mm	75	van Rijn et al, 2006[28]	70	79	Zauber et al, 2008[29]
Sensitivity for adenomas 6–9 mm	85	van Rijn et al, 2006[28]	80	92	Zauber et al, 2008[29]
Sensitivity for adenomas ≥10 mm	95	van Rijn et al, 2006[28]	93.1	99.5	Johnson et al, 2008[30]
Sensitivity for colorectal cancer	95	By assumption	93.1	99.5	By assumption
Reach††	95 to end of cecum, remainder between rectum and cecum	By assumption	Not varied	Not varied	
Sigmoidoscopy (within reach)					
Specificity†	87**	Weissfeld et al, 2005[31]	Not varied	Not varied	
Sensitivity for adenomas <6 mm	75	By assumption	70	79	
Sensitivity for adenomas 6–9 mm	85	By assumption	80	92	
Sensitivity for adenomas ≥10 mm	95	By assumption	93.1	99.5	
Sensitivity for colorectal cancer	95	By assumption	93.1	99.5	
Reach	76–88 to sigmoid-descending junction; 0 beyond the splenic flexure	Atkin et al, 2002[32] Painter et al, 1999[33]	Not varied	Not varied	

FIT = fecal immunochemical test; gFOBT = sensitive guaiac-based fecal occult blood test; mtSDNA = multitarget stool DNA test.

* Sensitivity estimates are per person for stool-based tests and per-lesion for endoscopic tests. Specificity is defined as the probability of a negative test result among persons who do not have adenomas or colorectal cancer.

^†^ Specificity is defined as the probability of a negative test result among persons who do not have adenomas or colorectal cancer.

^‡^ Sensitivity for persons with non-advanced adenomas. For persons with <6 mm adenomas, we assume that the sensitivity of the test is equal to the positivity rate in persons without adenomas (i.e., 1 –specificity). The sensitivity for persons with 6–9 mm adenomas is chosen such that the weighted average sensitivity for persons with <6 mm and with 6–9 mm adenoma(s) is equal to that of non-advanced adenomas.

^§^ Sensitivity for persons with advanced adenomas (i.e., adenomas ≥10 mm and/or adenomas with advanced histology). Sensitivity was not reported for the subset of ≥10 mm adenomas.

^‖^ We assume that <6 mm adenomas do not bleed, and therefore cannot cause a positive stool test. We also assume that gFOBT can be positive due to bleeding from other causes, the probability of which is equal to positivity rate in persons without adenomas (i.e., 1–0.925).

^¶^ We assume the same test characteristics for screening colonoscopies as for colonoscopies for diagnostic follow-up or for surveillance. We assume no correlation in findings between sigmoidoscopy and subsequent diagnostic colonoscopy.

** The lack of specificity with endoscopy reflects the detection of non-adenomatous polyps, which, in the case of sigmoidoscopy, may lead to unnecessary diagnostic colonoscopy, and in the case of colonoscopy screening, leads to unnecessary polypectomy, which is associated with an increased risk complications.

^††^ We assume that 5% of persons undergoing colonoscopy require 2 procedures to achieve complete visualization and that the cecum is ultimately visualized in 95% of patients.

### Costs

The analysis was conducted from the CMS perspective, and as such, costs were valued by Medicare reimbursement rates and excluded beneficiary copayments and cost-sharing payments. Costs of stool-based screening tests were based on the 2017 Clinical Diagnostic Laboratory Fee Schedule and costs of endoscopic tests were based on 2014 average Medicare payments (Table 2). For a detailed description of the derivation of these costs, see S1 Appendix. Endoscopy payments were updated to 2017 dollars using the Personal Health Care Deflator Price Index [34]. For each type of colonoscopy complication, the average payment by CMS was calculated using frequency data on hospitalizations for colonoscopy complications (personal communication, Craig Parzynski, MS, of Yale University).

**Table 2:** Reimbursement for screening tests and for colonoscopy complications, and annual reimbursements for cancer care used in the analysis.

Description				Reimbursement* ($)
**Screening tests**				
mtSDNA				512
FIT				22
gFOBT				4
Colonoscopy, without polypectomy, by indication				
- Screening				735
- Diagnostic				639
- Surveillance				724
Colonoscopy, with polypectomy				870
Sigmoidoscopy				337
**Colonoscopy complications**				
Serious GI complication (perforations, GI bleeding, transfusions)				6,847
Other GI complication (paralytic ileus, nausea and vomiting, dehydration, abdominal pain)				4,878
Cardiovascular complication (myocardial infarction or angina, arrhythmias, congestive heart failure, cardiac or respiratory arrest, syncope, hypotension, or shock)				5,347
**Cancer care**				
		**Annual reimbursement by stage at diagnosis ($)**		
**Phase of care**†	**I**	**II**	**III**	**IV**
Initial phase	33,341	47,283	67,300	97,931
Continuing phase	2,685	3,266	5,258	26,474
Terminal phase, non-CRC death	16,701	17,963	24,990	61,238
Terminal phase, CRC death	68,339	77,098	79,770	99,255

CRC = colorectal cancer; FIT = fecal immunochemical test; gFOBT = sensitive guaiac-based fecal occult blood test; GI = gastrointestinal; mtSDNA = multitarget stool DNA test.

* All costs are expressed in 2017 US dollars. Costs of stool-based tests are based on the 2017 Centers for Medicare and Medicaid Services Clinical Laboratory Fee Schedule. Costs of endoscopic procedures are based on 2014 average Medicare payments for a screening sigmoidoscopy and for each type of colonoscopy and include payments for pathology and anesthesia services.

^†^ The initial phase of care is the first 12 months after diagnosis, the last year of life phase is the final 12 months of life, and the continuing phase is all the months between the initial and last year of life phases.

Net costs of CRC-related care by stage at diagnosis and phase of care were obtained from an updated analysis of 2007–2013 SEER-Medicare linked data ([35] and personal communication, Angela Mariotto, PhD) and were updated to 2017 dollars (Table 2).

### Cost-effectiveness analysis

We used the simulation models to calculate lifetime costs of CRC screening and related care and life expectancy for a previously unscreened cohort of 65-year-old Medicare beneficiaries under eight CRC screening strategies, including no screening. We conducted an incremental cost-effectiveness analysis from the perspective of CMS and discounted both future costs and life-years 3% annually to account for time preference for present over future outcomes [36]. Screening strategies were ranked by increasing costs. Strategies that were more costly and less effective than another strategy (i.e., strongly dominated strategies) were eliminated from consideration because they were inefficient screening options. Of the remaining strategies, those that were less costly and less effective than another but provided an additional life-year gained (LYG) at a higher incremental cost (i.e., weakly dominated strategies) were also eliminated from consideration. The relative performance of the remaining non-dominated strategies was measured using the incremental cost-effectiveness ratio (ICER), defined as the additional cost of a specific strategy, divided by its additional clinical benefit (in this case, LYG), compared with the non-dominated strategy with costs closest to, but lower than, the strategy of interest.

All non-dominated strategies represent the set of potentially cost-effective options and together comprise the efficient frontier. Which strategy is ultimately deemed to be cost effective depends on the willingness to pay for a LYG. Although there is no official willingness-to-pay (WTP) threshold in the US, a strategy with an ICER less than $50,000–100,000 per LYG is generally considered to provide good value [37]. In this analysis, we assumed a WTP threshold of $100,000 per LYG.

### Outcomes

Outcomes are reported as the results predicted by each of the three models, focusing on the median prediction, along with estimates from the other two models, which define the range across models.

### Sensitivity analyses

Although this analysis was primarily performed to inform reimbursement decisions for Medicare beneficiaries, we included a sensitivity analysis in which we simulated a 50-year-old cohort.

We also explored the cost-effectiveness of mtSDNA testing using quality-adjusted life years (QALYs) gained as the measure of effectiveness. The assumptions for utilities are provided in S3 Table.

### Threshold analyses

If the triennial mtSDNA test strategy was found to be dominated by other screening options, we calculated the maximum cost per mtSDNA test (i.e., the threshold cost) for that strategy to be on the efficient frontier (i.e., to be potentially cost effective). If the mtSDNA test strategy was the least effective strategy, we calculated the threshold cost at which its total discounted cost would be $1 less than the total cost of the least expensive strategy on the efficient frontier (prior to the addition of the mtSDNA test strategy). If the mtSDNA test strategy was the most effective strategy, we calculated the threshold cost at which its ICER was <$100,000 per LYG. If the mtSDNA test strategy was between the least and most effective strategies on the efficient frontier, we calculated the threshold cost at which its ICER was equal to the ICER of the efficient strategy with LYG closest to and higher than the LYG of the mtSDNA test strategy.

Since the availability of the mtSDNA test could entice a previously unscreened individual to undergo screening, we also identified the threshold mtSDNA test cost for scenarios in which the adherence of the mtSDNA strategy was greater than that of all other screening strategies. For this sensitivity analysis we assumed a fixed overall adherence for each test [38], where those that adhere are completely adherent to screening and those who do not are completely non-adherent. We then increased the relative adherence for the mtSDNA test strategy from 0 to 40% higher than with other tests and calculated the associated threshold mtSDNA test costs. Note that simulating adherence in this manner means that the threshold costs do not change with the overall level of adherence of all other screening strategies, only with the relative increase in adherence with mtSDNA testing.

Threshold costs for mtSDNA were also identified in sensitivity analyses with: higher and lower estimates of the sensitivity of either mtSDNA testing or of all other screening modalities (Table 1); and annual and biennial screening intervals for mtSDNA testing.

Threshold costs are only influenced by the WTP threshold if mtSDNA testing is the most effective strategy. For those threshold analyses, we also present the threshold cost for WTP thresholds of $50,000 and $150,000 per LYG.

## Results

In the absence of screening, 64 (range across models: 61–64) per 1,000 65-year-olds will be diagnosed with CRC in their lifetimes (Table 3), resulting in approximately $4.0 million (range: $3.9–4.1 million) in discounted lifetime direct medical costs. All screening strategies yielded large reductions in CRC incidence and mortality. Assuming 100% adherence, the reduction in lifetime risk of CRC with one of the established screening strategies ranged from 50% (range: 36–59%) with annual FIT screening to 73% (range: 58–86%) with 10-yearly colonoscopy screening (S1 Fig, **Panel A**). With 46% (range: 33–54%) CRC risk reduction, triennial mtSDNA testing was less effective. Reductions in lifetime risk of CRC death (S1 Fig, **Panel B**) were higher than reductions in incidence but followed a similar pattern. The reduction in lifetime risk of CRC death ranged from 66% (range: 62–68%) with triennial mtSDNA testing to 84% (range: 80–92%) with 10-yearly colonoscopy screening.

**Table 3:** Undiscounted colorectal cancer cases and deaths, and discounted costs and life-years gained with associated incremental cost-effectiveness ratio of no colorectal cancer screening and seven colorectal cancer screening strategies in a cohort of 1,000 previously unscreened 65-year-olds, by model.

Strategy	CRC-SPIN					MISCAN					SimCRC				
	CRC cases	CRC deaths	Lifetime costs,* million $	LYG*	ICER, $	CRC cases	CRC deaths	Lifetime costs,* million $	LYG*	ICER, $	CRC cases	CRC deaths	Lifetime costs,* million $	LYG*	ICER, $
No screening	64	23	3.928	0	D	61	25	3.966	0	D	64	25	4.086	0	D
gFOBT 1y	25	6	2.387	89.5	--	39	8	3.599	86.6	D	31	7	3.134	91.6	D
FIT 1y	27	6	2.485	88.3	D	39	8	3.561	87.2	--	32	7	3.131	91.9	--
SIG 5y	29	9	3.110	70.8	D	30	7	3.878	88.9	D	28	9	3.603	80.1	D
SIG 10y + gFOBT 1y	17	4	2.489	99.0	D	29	6	3.747	98.7	16,200	23	5	3.282	99.1	20,900
SIG 10y + FIT 1y	17	4	2.581	98.5	D	29	6	3.782	99.0	D†	23	5	3.320	99.3	D†
COL 10y	9	2	2.479	107.4	5,100	25	5	3.846	101.6	34,700	17	4	3.406	102.8	33,200
mtSDNA 3y	30	8	3.887	79.3	D	41	9	4.889	81.7	D	34	8	4.512	87.9	D

-- = default strategy (i.e., the least costly and least effective non-dominated strategy); COL = colonoscopy; CRC = colorectal cancer; D = dominated; FIT = fecal immunochemical test; gFOBT = high sensitivity guaiac-based fecal occult blood test; ICER = incremental cost-effectiveness ratio; LYG = life-years gained compared with no screening; mtSDNA = multitarget stool DNA test; SIG = flexible sigmoidoscopy.

* Future costs and life-years are discounted at a 3% annual rate.

^†^ Indicates a dominated strategy is weakly dominated (i.e., one of the other strategies provides more life-years gained than this strategy, and it has a lower incremental cost-effectiveness ratio). All other dominated strategies are strongly dominated (i.e., provide fewer life-years gained and have higher total costs than another strategy).

### Cost-effectiveness analysis

**Fig 1:**
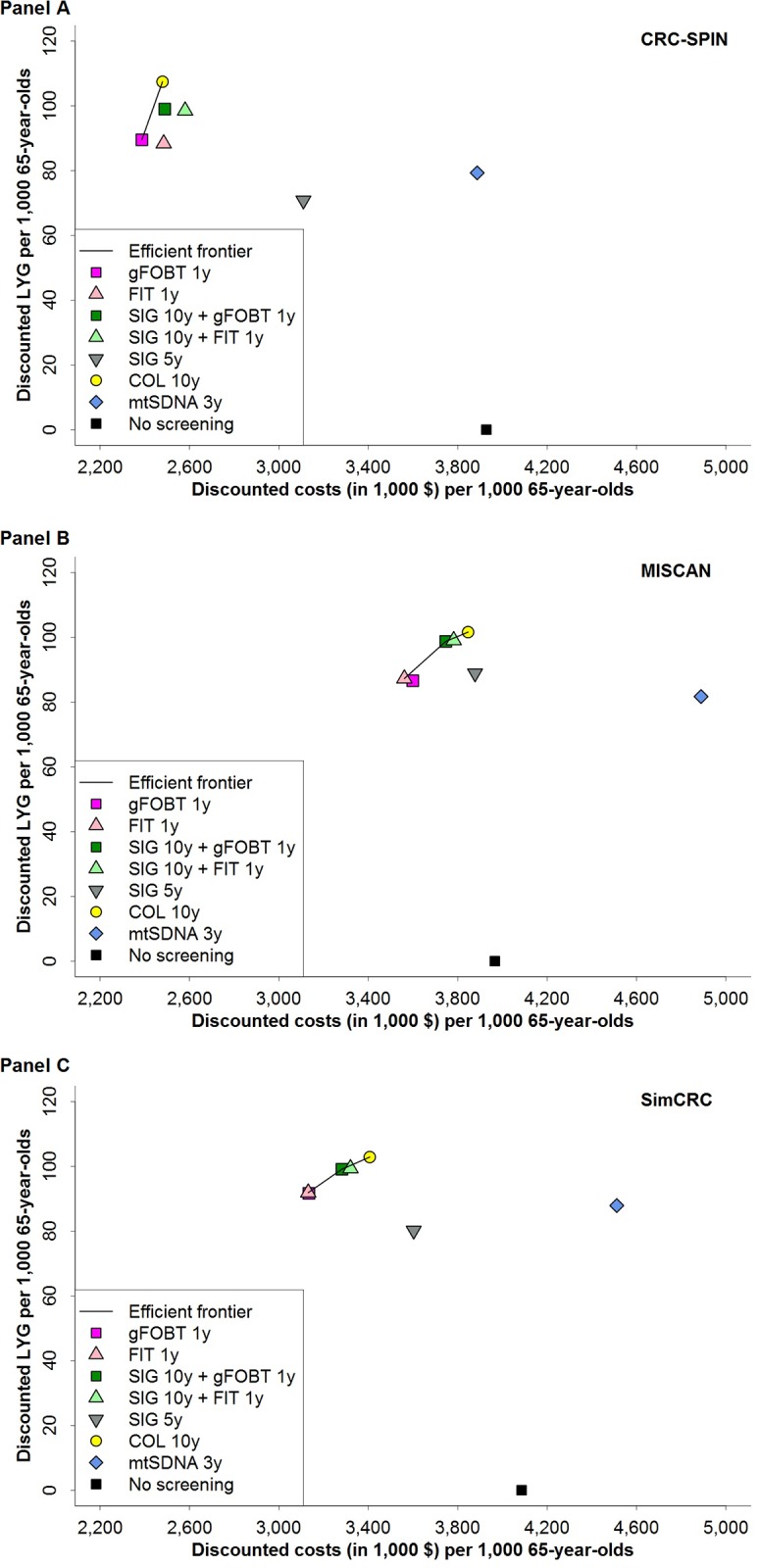
Discounted costs and discounted life-years gained per 1,000 persons aged 65 years for eight colorectal cancer screening strategies and the efficient frontier connecting the economically efficient strategies, for CRC-SPIN (Panel A), MISCAN (Panel B) and SimCRC (Panel C) models. Discounted costs and life-years gained reflect total costs and life-years gained of a screening program, accounting for time preference for present over future outcomes. Life-years gained are plotted on the *y*-axis, and total costs are plotted on the *x*-axis. Each possible screening strategy is represented by a point. Strategies that form the solid line connecting the points lying left and upward are the economically rational subset of choices. This line is called the *efficient frontier*. The inverse slope of the line represents the incremental cost-effectiveness ratio of the connected strategies. Points lying to the right and beneath the line represent the dominated strategies. Screening with the multitarget stool DNA test every 3 years has higher costs and fewer life-years gained than screening annually with either gFOBT or FIT, and the multitarget stool DNA strategy is therefore strongly dominated. COL = colonoscopy; FIT = fecal immunochemical test; gFOBT = guaiac-based fecal occult blood test; LYG = life-years gained; mtSDNA = multitarget stool DNA test; SIG = flexible sigmoidoscopy.

Two screening strategies were found to be efficient by all models: 10-yearly colonoscopy and annual FOBT using either gFOBT or FIT (Fig 1, Table 3). Ten-yearly sigmoidoscopy combined with annual gFOBT was also an efficient screening option in two models (MISCAN and SimCRC).

In one model (MISCAN), triennial mtSDNA testing yielded the fewest LYG of all evaluated strategies, and in the other two models it had the second-fewest LYG after 5-yearly sigmoidoscopy. Two models (MISCAN and SimCRC) found that triennial mtSDNA testing was the most expensive strategy evaluated; one model (CRC-SPIN) found that only no screening was more expensive than mtSDNA testing. Given the limited LYG and higher costs, triennial mtSDNA testing was not an efficient alternative to other strategies reimbursed by CMS.

### Sensitivity analyses

Simulating a 50-year-old cohort or using QALYs as the measure of effectiveness did not change the conclusions about the comparative effectiveness and cost-effectiveness of the different strategies (S4 **and** S5 Tables, **respectively**). Triennial mtSDNA testing yielded either the fewest (MISCAN) or second-fewest (CRC-SPIN and SimCRC) LYG; and in two models (MISCAN, SimCRC) mtSDNA testing was the most expensive strategy, while in one (CRC-SPIN) only no screening was more costly.

### Threshold analyses

**Fig 2:**
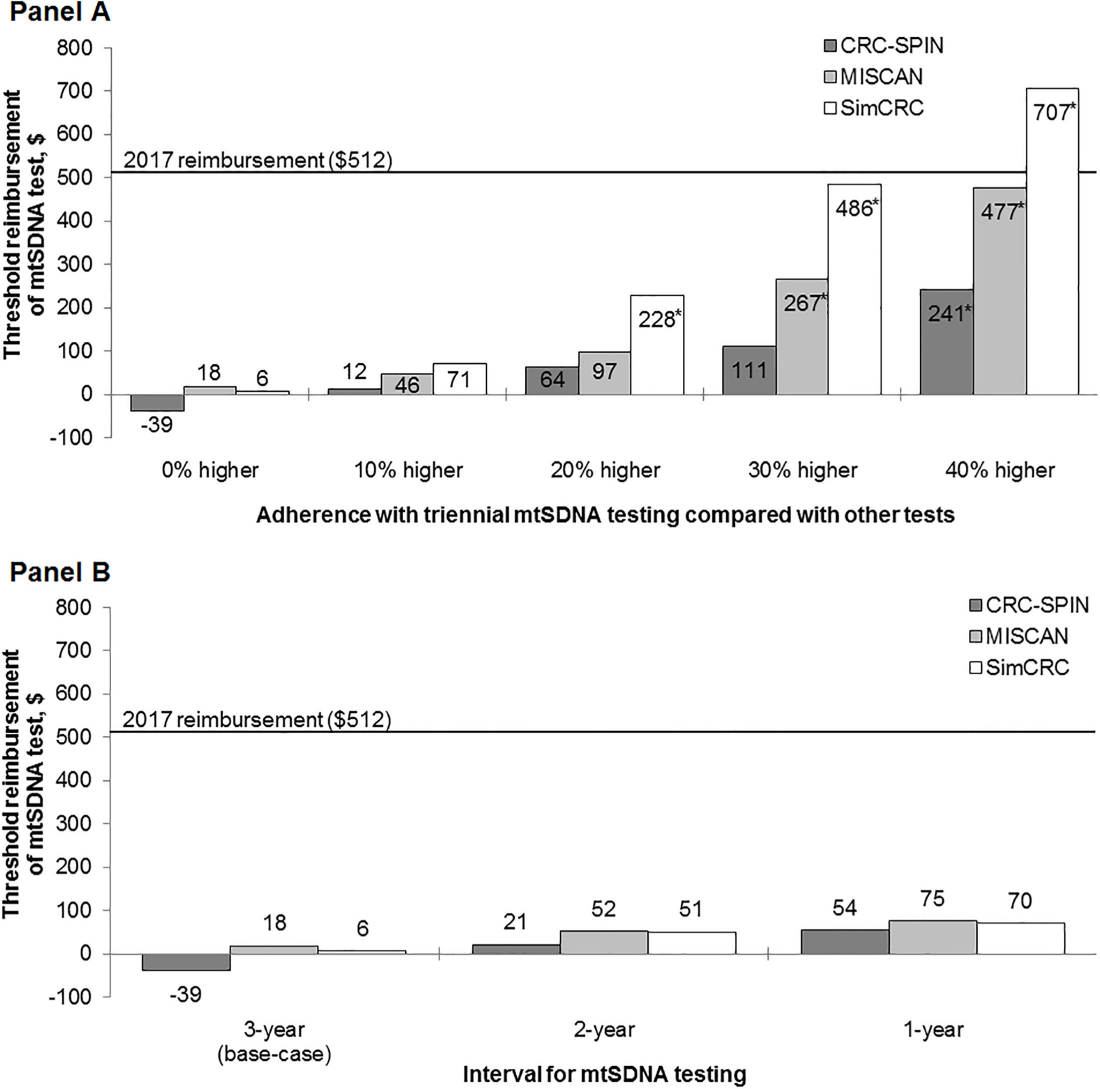
Sensitivity analyses: Reimbursement thresholds for the mtSDNA test at which the mtSDNA test strategy is efficient compared with other reimbursed CRC screening strategies for different levels of adherence with the mtSDNA strategy (Panel A) and for different intervals of screening with the mtSDNA test (Panel B). mtSDNA = multitarget stool DNA test. * For these adherence levels, the threshold cost of mtSDNA test was dependent on the willingness-to-pay threshold. S3 Fig shows the threshold costs for willingness-to-pay thresholds of $50,000 and $150,000 per life year gained.

In threshold analyses, two models (MISCAN and SimCRC) found that the reimbursement for the mtSDNA test must be considerably lower, in the range of $6–18 per test, for triennial mtSDNA screening to be an efficient and potentially cost-effective strategy (Fig 2). In one model (CRC-SPIN), there was no level of reimbursement at which triennial mtSDNA testing would be cost effective compared with currently recommended screening options (i.e., the threshold cost was negative), due to the benefit being low relative to other tests.

If the triennial mtSDNA test strategy would motivate individuals who would not otherwise be screened to participate in screening, then the threshold cost at which the mtSDNA strategy would be on the efficient frontier would increase. Adherence with mtSDNA testing would need to be 31–53% better than with other tests in order for triennial mtSDNA testing to be efficient at the base-case reimbursement rate of $512 (Fig 2, **Panel A**).

If mtSDNA was assumed to perform better at detecting disease or if other modalities were assumed to perform worse than assumed in the base-case analysis, estimated reimbursement rates increased to $32–42 (one model (CRC-SPIN) continued to find no reimbursement rate at which the mtSDNA test strategy would be an efficient option) and $40 (range: $1–52), respectively (S2 Fig). When the interval of mtSDNA screening was shortened to 2 years or 1 year, threshold costs increased to $51 (range: $21–52) and $70 (range: $54–75), respectively (Fig 2, **Panel B**).

The WTP threshold only influenced the threshold cost of the mtSDNA test when we assumed that adherence with mtSDNA testing would be higher than with other strategies. For SimCRC the threshold cost is affected by the WTP threshold at ≥17% higher adherence, for MISCAN the threshold cost is affected at ≥24% higher adherence, and for CRC-SPIN the threshold cost is affected at ≥36% higher adherence (S3 Fig).

## Discussion

This study showed that, with perfect adherence, a 10-year period of triennial mtSDNA screening in previously unscreened 65-year-olds was slightly less effective in terms of LYG than a 10-year period of annual FIT screening. At its 2017 reimbursement rate of $512 per test, triennial mtSDNA testing was the most costly strategy and was therefore not cost effective compared to other screening options available to Medicare beneficiaries. Per-test reimbursement for the mtSDNA test would need to be less than $6–18 (or even negative, according to one model) for triennial screening to be potentially cost effective compared to other reimbursed strategies. Higher reimbursement rates could be supported with more frequent screening: $51 (range: $21–52) per test with biennial screening and $70 (range: $54–75) per test with annual screening.

These results show that if a previously unscreened 65-year-old were to choose between triennial mtSDNA testing and another reimbursed strategy, it would be both more effective and more cost effective to choose the other strategy. However, mtSDNA testing does reduce CRC incidence and mortality compared to no screening at all. Therefore, mtSDNA testing could be cost effective if it were reserved solely for use by those who are unwilling to participate in CRC screening with any other available test. However, this test has been marketed to the US public for general use, and a study on early adoption of mtSDNA testing found higher usage of mtSDNA among patients with prior CRC screening compared to those without [39].

Despite its higher sensitivity for advanced adenomas and cancer, triennial mtSDNA testing yielded fewer LYG than the two other stool-based CRC screening strategies evaluated. The lower effectiveness can be explained by the longer screening interval. For example, consider a simple case of a person with a single advanced adenoma: if tested with annual FIT, the probability of a positive test is 0.558 after 3 annual screens [i.e., 1—(1–0.238)^3^], if screening results are independent within an individual. If tested with triennial mtSDNA, there would be only one opportunity for detection in the 3-year period, with a probability of a positive test equal to 0.424. In summary, if tests are independent within individuals then a program of annual FIT is both more sensitive and more specific than triennial mtSDNA. If performed annually or biennially, programmatic sensitivity of mtSDNA screening would increase, but this would likely come at the cost of increased false-positive test results. Therefore, threshold costs only showed a modest increase when the interval was shortened.

When reimbursed at $512 per test, mtSDNA could only be cost effective compared to other strategies if adherence with mtSDNA were considerably higher (31–53% higher) than adherence with other tests. The mtSDNA test may appeal to some unscreened persons because it is non-invasive, has higher sensitivity (but lower specificity) than other stool tests and can be performed less frequently. However, it is still a stool test (and in fact, requires patients to sample from the collected stool for the immunochemical assay portion of the test), and, as such, the demonstrated barriers to this form of screening, such as handling of stool and storing stool in the house for a short period of time [40] also apply to mtSDNA testing. Furthermore, mtSDNA does not eliminate the barriers common to all screening tests, namely financial barriers, failure of clinicians to advise about CRC screening, and not knowing testing was necessary [40].

CMS’s high reimbursement rate for Cologuard—more than 20 times the reimbursement for other stool-based screening tests for CRC—is the result of federal regulations for setting reimbursement for new diagnostic laboratory tests. Payment for a new diagnostic laboratory test is set by one of two approaches: “cross-walking” or “gap-filling” [41]. Cross-walking is used if the new test is comparable to one or more existing tests already reimbursed under the Clinical Laboratory Fee Schedule (CLFS); reimbursement for the new test is set equal to the reimbursement for the comparable test(s). If no comparable test exists, then payment for a new diagnostic laboratory test is set using the gap-filling approach. With that approach, other information is taken into consideration, including information on reimbursement for the test in non-Medicare settings and resource use required for other relevant tests. Reimbursement for Cologuard was set by cross-walking to three existing codes on the CLFS (81315, 81275, and 82274), yielding the 2014 reimbursement of $492.72 [42]. Had CMS instead used the gap-filling approach, it is possible that CRC screening with Cologuard would have been a cost-effective alternative to other tests reimbursed by CMS. Reimbursement for clinical laboratory tests, including gFOBT, FIT, and Cologuard, changed when Section 216 of the Protecting Access to Medicare Act of 2014 went into effect on January 1, 2018 [43]. In 2019, the reimbursement for gFOBT, FIT, and Cologuard were $4.38, $17.67, and $508.87, respectively.

Our findings are in line with our previous analysis of the cost-effectiveness of stool DNA testing, in which we considered a hypothetical test called “sDNA version 2.0” [44, 45]. This test had sensitivity and specificity similar to the mtSDNA test, but was never available to the public. For that test, we found threshold reimbursement rates of $17–41, which are similar to the current estimates.

A recent modeling study by Ladabaum and Mannalithara [46] also showed that mtSDNA testing is not cost effective compared to other screening modalities, unless mtSDNA screening resulted in higher participation rates or would cost less than its current reimbursement rate. Our study incorporates three models that all consistently show that adding mtSDNA screening to the menu of CRC screening options results in even lower value than estimated by Ladabaum and Mannalithara. We found that mtSDNA screening is not cost effective even under extremely unrealistic assumptions of a 30% higher adherence than with other tests or test costs of $70. The most likely reason for the difference in threshold costs and adherence, is that Ladabaum and Mannalithara performed their threshold analysis for very specific situations, with either $153 for patient navigation costs added to the cost of FIT, or assuming that only 45% of individuals would ever participate in FIT-based screening.

An important strength of the current study is the use of three independently developed models. Some limitations are noteworthy. First, the models assume that all CRCs arise through the traditional adenoma–carcinoma sequence, and none incorporate a separate pathway for sessile serrated adenomas. However, for both FIT and mtSDNA, the sensitivity for detecting advanced adenomas includes both traditional advanced and sessile serrated adenomas [14]. Our models would underestimate the effectiveness of mtSDNA compared to FIT only if mtSDNA plus diagnostic colonoscopy would detect more serrated adenomas than FIT plus diagnostic colonoscopy *and* these lesions have higher malignant potential than traditional advanced adenomas. There is evidence to suggest that FIT might be less sensitive than mtSDNA for sessile serrated adenomas [14, 47], but their malignant potential seems comparable to that of traditional advanced adenomas [48]. Therefore, the impact on (cost-)effectiveness outcomes of not having two separate pathways to CRC in our models is expected to be limited.

Second, the models simulate the progression from adenoma to CRC by allowing adenomas to increase in size over time. Because adenoma size and presence of villous components or high-grade dysplasia are highly correlated [49], size indirectly represents histology and grade. However, none of the models separately simulate the step from adenoma with low-grade dysplasia to adenoma with high-grade dysplasia. For the sensitivity of mtSDNA to detect large adenomas (≥1 cm), we used the estimate for advanced adenomas from Imperiale et al. [14], who defined advanced adenomas as those with high-grade dysplasia or ≥25% villous histologic features or measuring ≥1 cm in size. As colonoscopy sensitivity increases with size, but not histology, of the adenoma, the follow-up colonoscopy after a positive mtSDNA test will detect more high-grade dysplasia and adenomas with villous components if they are all assumed to be of large size, as opposed to if we had modeled histology and grade explicitly. As a result, we may have overestimated the effectiveness of mtSDNA testing.

Third, the simulated cohort did not have any CRC screening prior to age 65. In practice, the Medicare population increasingly consists of individuals who have already had some type of CRC screening. Although the value of screening is lower for individuals with prior screening (because they have lower disease prevalence), the relative difference in effectiveness and cost-effectiveness between different screening strategies is expected to be similar.

Fourth, we assumed conditional independence of repeat screenings. Consequently we assumed that there were no systematic false-negative results for adenomas and cancers. This assumption may not hold for gFOBT and FIT testing because bleeding of a lesion may not be a random event [50]. However, this assumption may not hold for mtSDNA testing either, because testing for blood is an important component of the test. Furthermore, the lesion in question may have acquired a gene mutation not assessed by the mtSDNA test, which means our assumption of conditional independence may be less likely to hold for the mtSDNA test compared to gFOBT and FIT. As a result, we may have overestimated the benefit of stool tests in general and of mtSDNA testing in particular, and its threshold reimbursement rate may be even lower than estimated here.

Finally, because test-specific data on longitudinal screening patterns are lacking, our base-case analysis assumes 100% adherence with screening, follow-up and surveillance procedures. Uptake of screening among the Medicare population is considerably less than 100% [38], as is adherence with repeat screening [51], follow-up [52] and surveillance [53]. Meanwhile, overuse of resources is also common [54], and a positive stool test is sometimes followed by another stool test instead of by the prescribed follow-up colonoscopy [55]. Although we did include a sensitivity analysis with lower uptake, we assumed that individuals were either fully adherent or fully non-adherent with screening. The impact of less-than-perfect adherence among those who take up screening will vary according to the interval of testing and the characteristics of the test. While stool-based testing may have higher initial acceptance rates,[56–59] patients’ willingness to comply with annual stool-based testing over longer periods of time is uncertain,[60–62] and no data is available on adherence with triennial mtSDNA testing.

In summary, our analysis shows that compared with no screening, triennial mtSDNA testing reduces CRC incidence and mortality. However, with perfect adherence, it is less effective than other CRC screening options available to Medicare beneficiaries. At its current reimbursement rate, triennial mtSDNA testing also has higher costs than all other strategies, making it an inefficient screening option. It could be efficient and potentially cost effective if mtSDNA testing would increase adherence with CRC screening to nearly 100%. Triennial (or more frequent) mtSDNA testing could also be potentially cost effective if the reimbursement rate were substantially lower, i.e. similar to that of other stool-based tests.

## Supporting information

S1 Fig Reductions in the lifetime risk of being diagnosed with (Panel A) and dying from (Panel B) colorectal cancer. Median reduction and the range across models are shown for each screening strategy. COL = colonoscopy; CRC = colorectal cancer; FIT = fecal immunochemical test; gFOBT = high sensitivity guaiac-based fecal occult blood test; mtSDNA = multitarget stool DNA test; SIG = flexible sigmoidoscopy.
(TIF)

S2 Fig Sensitivity analyses: Reimbursement thresholds for the mtSDNA test at which the mtSDNA test strategy is efficient compared with other reimbursed CRC screening strategies for different levels of the sensitivity of the mtSDNA test (Panel A), and for different levels of the sensitivity of all other tests (Panel B). mtSDNA = multitarget stool DNA test. * See Table 1 for the worst-case, base-case, and best-case test sensitivities for mtSDNA and all other tests.
(TIF)

S3 Fig Sensitivity analyses: Reimbursement thresholds for the mtSDNA test at which the mtSDNA test strategy is efficient compared with other reimbursed CRC screening strategies for different levels of adherence with the mtSDNA strategy relative to other strategies, assuming a willingness-to-pay threshold of $50,000 per life-year gained (Panel A) and $150,000 per life-year gained (Panel B). mtSDNA = multitarget stool DNA test.
(TIF)

S1 Table Current Procedural Terminology codes and Healthcare Common Procedure Coding System codes used for colorectal cancer screening, follow-up, and surveillance tests and procedures, and mean reimbursement for the test/procedure and accompanying pathology and anesthesia services [1–3]. COL = colonoscopy; CPT code = Current Procedural Terminology code; FIT = fecal immunochemical test; gFOBT = guaiac-based fecal occult blood test; HCPCS code = Healthcare Common Procedure Coding System code; mtSDNA = multitarget stool DNA test. * Includes facility payments, when appropriate. † Reimbursement for pathology services was assumed to apply only to colonoscopy procedures in which polypectomy was performed. ‡ Sigmoidoscopy is simulated without biopsy or polypectomy of detected lesions. § If multiple polyps are removed by different methods (S2 Table), more than one CPT code may be submitted for one colonoscopy. In such cases, payment for all but the highest-reimbursed procedure is reduced to the difference between the payment for the procedure of interest and the payment for basic washing of the colon (CPT 45378).
(DOCX)

S2 Table Descriptions of the surgical Current Procedural Terminology codes used for colonoscopies with biopsy or polypectomy. CPT code = Current Procedural Terminology code. * Code was deleted in 2015 and replaced with 45388.
(DOCX)

S3 Table Utility decrements used in the sensitivity analysis with quality-adjusted life years as the measure of effectiveness. These decrements were applied to age-based utility weights for general health [1]. CRC = colorectal cancer; FIT = fecal immunochemical test; gFOBT = sensitive guaiac-based fecal occult blood test; GI = gastrointestinal; LY = life year; mtSDNA = multitarget stool DNA test. * Disutility of colonoscopy (0.12) is from Swan et al. [2]. Duration of disutility (1.5 days, or 0.41% of a year) is from Jonas et al. [3]. † Disutility of sigmoidoscopy was assumed to be the same as for colonoscopy. Duration of disutility was assumed to be 0.4 days, 0.12% of a year. ‡ Disutility of complications was assumed to be 0.5. Duration of disutility was assumed to be 4 days, 2 days, and 3.5 days for serious GI, other GI, and cardiovascular complications respectively. § The initial phase of care is the first 12 months after diagnosis, the last year of life phase is the final 12 months of life, and the continuing phase is all the months between the initial and last year of life phases. Disutilities per LY with CRC were derived from Ness et al. [4].
(DOCX)

S4 Table Undiscounted colorectal cancer cases and deaths, and discounted costs and life-years gained with associated incremental cost-effectiveness ratio of no colorectal cancer screening and seven colorectal cancer screening strategies in a cohort of 1,000 previously unscreened 50-year-olds, by model. **—** = default strategy (i.e., the least costly and least effective non-dominated strategy); COL = colonoscopy; CRC = colorectal cancer; D = dominated; FIT = fecal immunochemical test; gFOBT = high sensitivity guaiac-based fecal occult blood test; ICER = incremental cost-effectiveness ratio; LYG = life-years gained compared with no screening; mtSDNA = multitarget stool DNA test; SIG = flexible sigmoidoscopy. * Future costs and life-years are discounted at a 3% annual rate. † Indicates a dominated strategy is weakly dominated (i.e., one of the other strategies provides more life-years gained than this strategy, and it has a lower incremental cost-effectiveness ratio). All other dominated strategies are strongly dominated (i.e., provide fewer life-years gained and have higher total costs than another strategy).
(DOCX)

S5 Table Discounted costs and quality-adjusted life-years gained with associated incremental cost-effectiveness ratio of no colorectal cancer screening and seven colorectal cancer screening strategies in a cohort of 1,000 previously unscreened 65-year-olds, by model. **—** = default strategy (i.e., the least costly and least effective non-dominated strategy); COL = colonoscopy; CRC = colorectal cancer; D = dominated; FIT = fecal immunochemical test; gFOBT = high sensitivity guaiac-based fecal occult blood test; ICER = incremental cost-effectiveness ratio; QALYG = quality-adjusted life-years gained compared with no screening; mtSDNA = multitarget stool DNA test; SIG = flexible sigmoidoscopy. * Future costs and quality-adjusted life-years are discounted at a 3% annual rate. † Indicates a dominated strategy is weakly dominated (i.e., one of the other strategies provides more quality-adjusted life-years gained than this strategy, and it has a lower incremental cost-effectiveness ratio). All other dominated strategies are strongly dominated (i.e., provide fewer quality-adjusted life-years gained and have higher total costs than another strategy).
(DOCX)

S1 Appendix Calculation of screening costs. (DOCX)
