# Supplementary figures and images for: Cost-effectiveness of a multitarget stool DNA test for colorectal cancer screening of Medicare beneficiaries
Source: PLoS One. 2019 Sep 4;14(9):e0220234. doi: 10.1371/journal.pone.0220234 (PMC6726189; doi:10.1371/journal.pone.0220234)

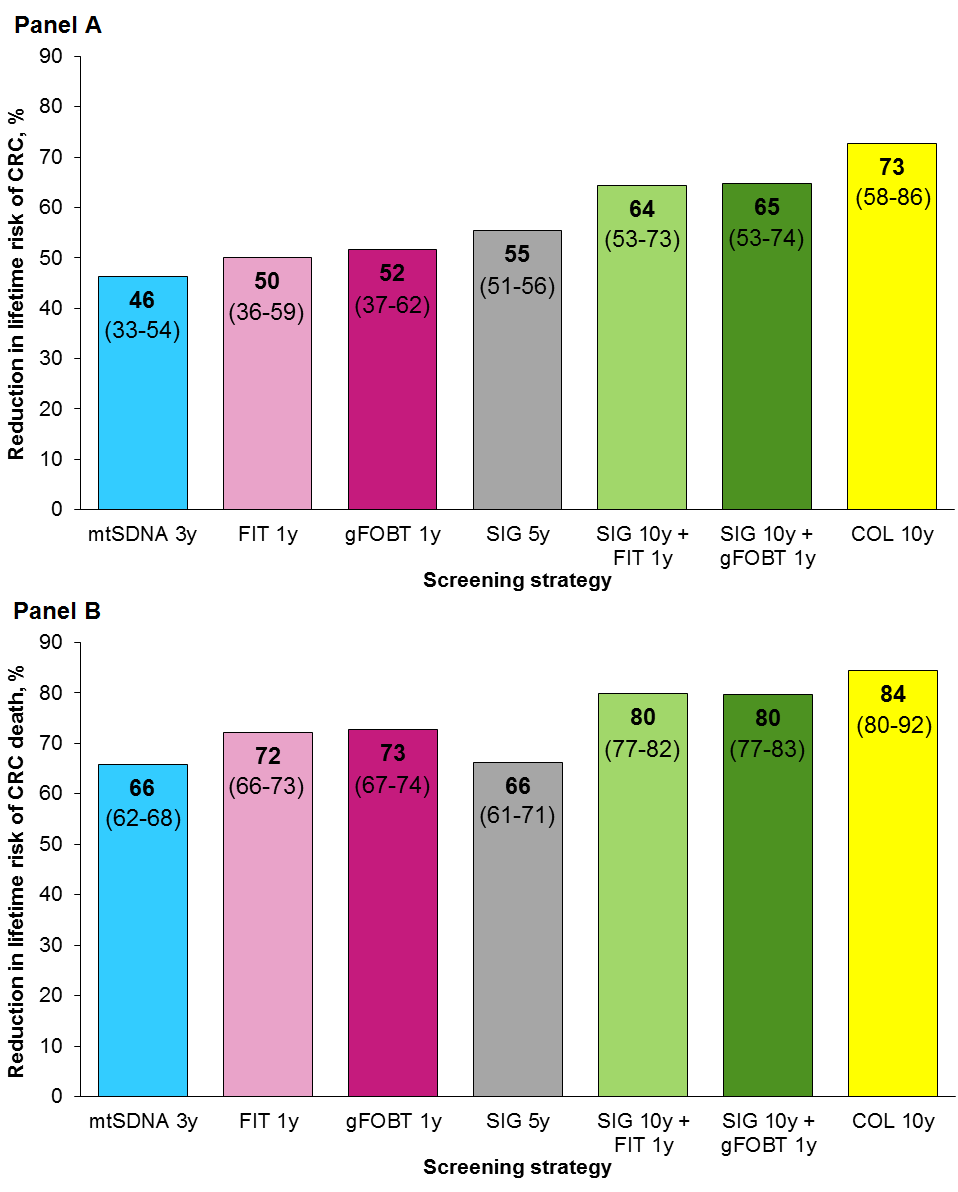

Supplement: S1 Fig — COL = colonoscopy; CRC = colorectal cancer; FIT = fecal immunochemical test; gFOBT = high sensitivity guaiac-based fecal occult blood test; mtSDNA = multitarget stool DNA test; SIG = flexible sigmoidoscopy. (TIF) [file pone.0220234.s001.tif]

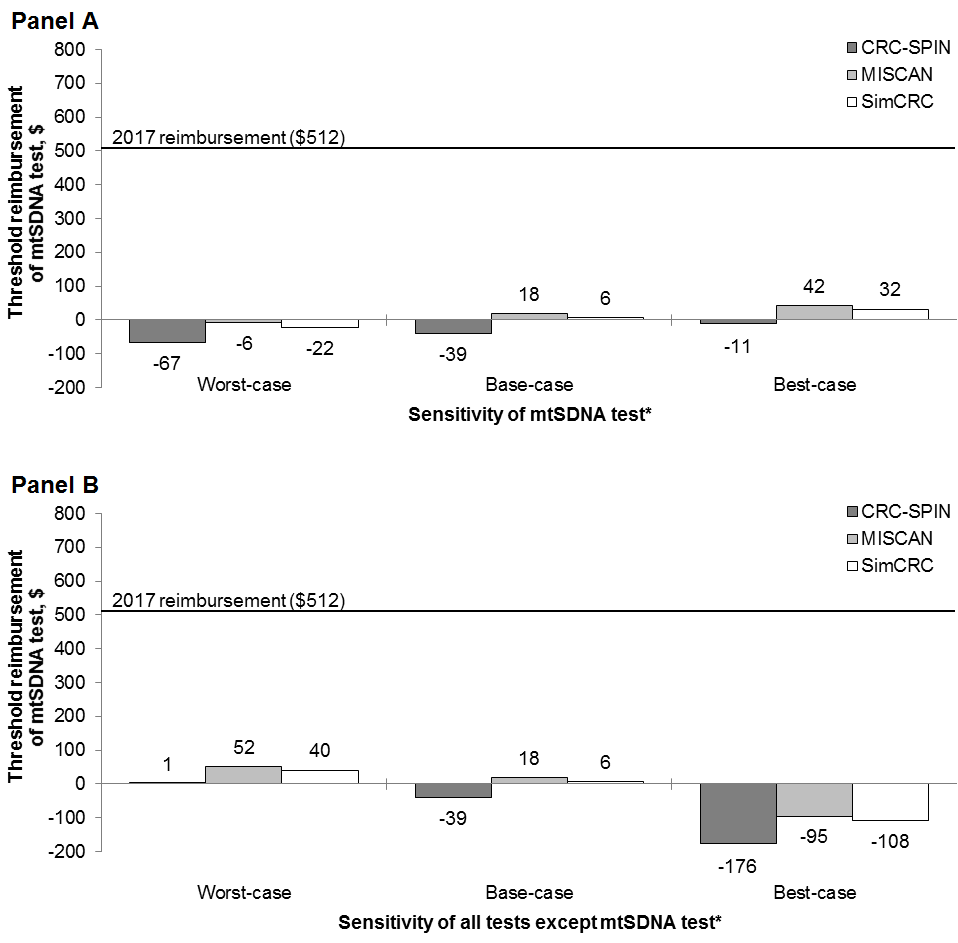

Supplement: S2 Fig — mtSDNA = multitarget stool DNA test. * See Table 1 for the worst-case, base-case, and best-case test sensitivities for mtSDNA and all other tests. (TIF) [file pone.0220234.s002.tif]

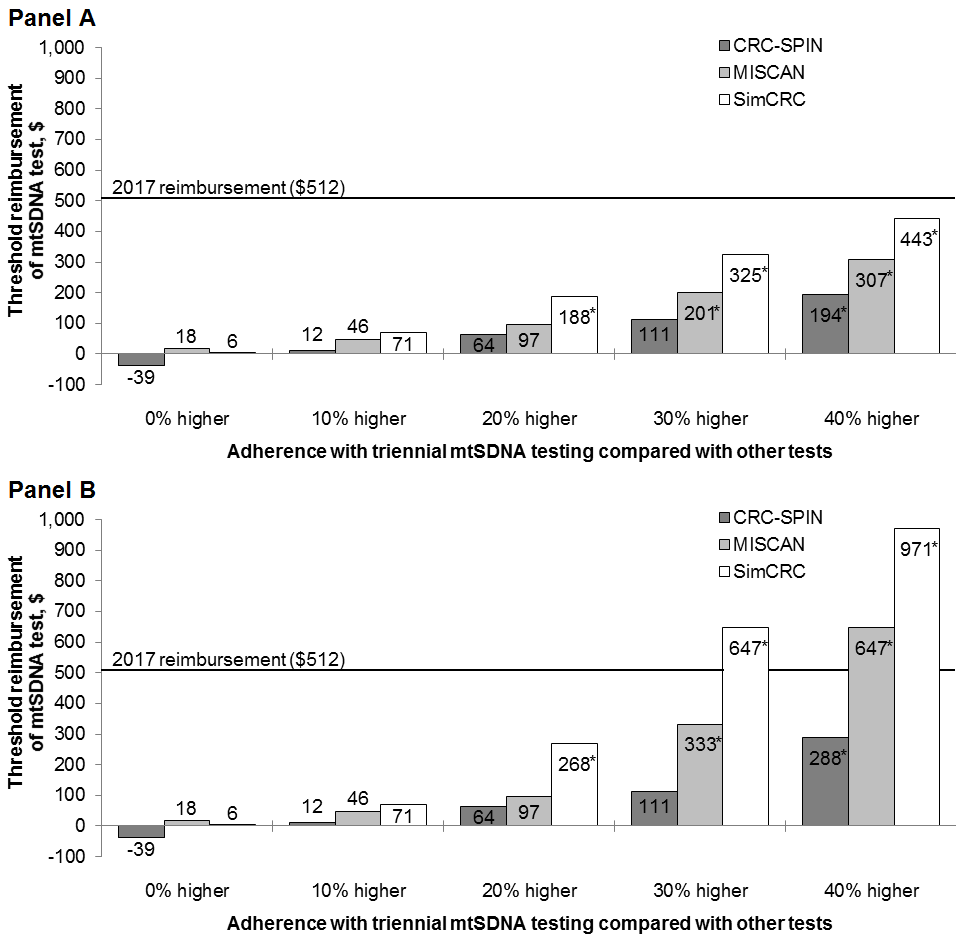

Supplement: S3 Fig — mtSDNA = multitarget stool DNA test. (TIF) [file pone.0220234.s003.tif]
