# Supplementary material for: Cost-effectiveness of a multitarget stool DNA test for colorectal cancer screening of Medicare beneficiaries
Source: PLoS One. 2019 Sep 4;14(9):e0220234. doi: 10.1371/journal.pone.0220234 (PMC6726189; doi:10.1371/journal.pone.0220234)
Supplement: S1 Table — COL = colonoscopy; CPT code = Current Procedural Terminology code; FIT = fecal immunochemical test; gFOBT = guaiac-based fecal occult blood test; HCPCS code = Healthcare Common Procedure Coding System code; mtSDNA = multitarget stool DNA test. * Includes facility payments, when appropriate. † Reimbursement for pathology services was assumed to apply only to colonoscopy procedures in which polypectomy was performed. ‡ Sigmoidoscopy is simulated without biopsy or polypectomy of detected lesions. § If multiple polyps are removed by different methods (S2 Table), more than one CPT code may be submitted for one colonoscopy. In such cases, payment for all but the highest-reimbursed procedure is reduced to the difference between the payment for the procedure of interest and the payment for basic washing of the colon (CPT 45378). (DOCX) [file pone.0220234.s004.docx]

|  |  |  | **Mean payment, by component** | | | |
| --- | --- | --- | --- | --- | --- | --- |
| **Test/procedure** | **CPT/HCPCS code** | **CPT/HCPCS description** | **Test/**  **Procedure*** | **Pathology payment†** | **Anesthesia services** | **Total** |
| gFOBT | 82270 | Guaiac-based fecal occult blood test | $4.46 | $0 | $0 | $4.46 |
| FIT | G0328 | Immunochemical fecal occult blood test | $21.82 | $0 | $0 | $21.82 |
| mtSDNA | G0464/81528 | Stool-based DNA and immunochemical fecal occult blood test | $512.43 | $0 | $0 | $512.43 |
| Sigmoidoscopy‡ | G0104 | Screening sigmoidoscopy | $301.89 | $0 | $33.84 | $336.72 |
| Screening COL without  lesion removal | G0121 | Screening colonoscopy; average risk | $671.31 | $0 | $51.68 | $734.51 |
| Follow-up COL (after positive finding on another screening test) without lesion removal | 45378 | Diagnostic colonoscopy for persons with signs/symptoms | $565.65 | $0 | $66.47 | $639.25 |
| Surveillance COL without lesion removal | G0105 | Screening colonoscopy; high-risk | $656.57 | $0 | $55.01 | $724.42 |
| Any COL with lesion removal§ | One or more of 45380-45381, 45383-45385 | Colonoscopy with intervention (hot/cold biopsy, snare biopsy, other) | $693.14 | $98.57 | $77.83 | $869.53 |

## References

1. Centers for Medicare and Medicaid Services. 2017 Clinical Laboratory Fee Schedule (CLAB) Public Use File (PUF). Available at https://www.cms.gov/Medicare/Medicare-Fee-for-Service-Payment/ClinicalLabFeeSched/Clinical-Laboratory-Fee-Schedule-Files-Items/17CLAB.html. 2017.

2. Chronic Condition Data Warehouse [cited 2016 January 13, 2016]. Available from: www.ccwdata.org.

3. https://www.cms.gov/Research-Statistics-Data-and-Systems/Statistics-Trends-and-Reports/NationalHealthExpendData/Downloads/Tables.zip, “Table 23. National Health Expenditures; Nominal Dollars, Real Dollars, Price Indexes, and Annual Percent Change: Selected Calendar Years 1980-2017” (row 41).
