# Supplementary material for: Cost-effectiveness of a multitarget stool DNA test for colorectal cancer screening of Medicare beneficiaries
Source: PLoS One. 2019 Sep 4;14(9):e0220234. doi: 10.1371/journal.pone.0220234 (PMC6726189; doi:10.1371/journal.pone.0220234)
Supplement: S2 Table — CPT code = Current Procedural Terminology code. * Code was deleted in 2015 and replaced with 45388. (DOCX) [file pone.0220234.s005.docx]

| **CPT code** | **Description** |
| --- | --- |
|  |  |
| 45380 | Colonoscopy with biopsy, single or multiple (forceps to grab tissue w/o cautery) |
| 45381 | Colonoscopy with submucosal injection |
| 45384 | Colonoscopy with removal of tumor(s), polyp(s), or other lesion(s) by hot biopsy forceps |
| 45385 | Colonoscopy with removal of tumor(s), polyp(s), or other lesion(s) by snare technique |
| 45383* | Colonoscopy with ablation of tumor(s), polyp(s) or other lesion(s) not amenable to removal by hot biopsy forceps, bipolar cautery or snare technique |
