# Supplementary material for: Cost-effectiveness of a multitarget stool DNA test for colorectal cancer screening of Medicare beneficiaries
Source: PLoS One. 2019 Sep 4;14(9):e0220234. doi: 10.1371/journal.pone.0220234 (PMC6726189; doi:10.1371/journal.pone.0220234)
Supplement: S3 Table — CRC = colorectal cancer; FIT = fecal immunochemical test; gFOBT = sensitive guaiac-based fecal occult blood test; GI = gastrointestinal; LY = life year; mtSDNA = multitarget stool DNA test. * Disutility of colonoscopy (0.12) is from Swan et al. [2]. Duration of disutility (1.5 days, or 0.41% of a year) is from Jonas et al. [3]. † Disutility of sigmoidoscopy was assumed to be the same as for colonoscopy. Duration of disutility was assumed to be 0.4 days, 0.12% of a year. ‡ Disutility of complications was assumed to be 0.5. Duration of disutility was assumed to be 4 days, 2 days, and 3.5 days for serious GI, other GI, and cardiovascular complications respectively. § The initial phase of care is the first 12 months after diagnosis, the last year of life phase is the final 12 months of life, and the continuing phase is all the months between the initial and last year of life phases. Disutilities per LY with CRC were derived from Ness et al. [4]. (DOCX) [file pone.0220234.s006.docx]

| **Description** |  | | | | | **Utility loss per event** | |
| --- | --- | --- | --- | --- | --- | --- | --- |
|  |  | | | | |  | |
| **Screening tests** | | | | | |  | |
| mtSDNA | | | | | | 0 | |
| FIT | | | | | | 0 | |
| gFOBT | | | | | | 0 | |
| Colonoscopy* | | | | | | 0.0005 | |
| Sigmoidoscopy† | | | | | | 0.0001 | |
| **Complications‡** | |  | | | |  | |
| Serious GI complication (perforations, GI bleeding, transfusions) | | | | | |  | 0.0055 |
| Other GI complication (paralytic ileus, nausea and vomiting, dehydration, abdominal pain) | | | | | |  | 0.0027 |
| Cardiovascular complication (myocardial infarction or angina, arrhythmias, congestive heart failure, cardiac or respiratory arrest, syncope, hypotension, or shock) | | | | | | 0.0048 | |
|  | |  | **Utility loss per LY with CRC, by stage at diagnosis** | | | | |
| **Phase of care§** | | | **I** | **II** | **III** | **IV** | |
|  | |  |  |  |  |  | |
| Initial phase | |  | 0.12 | 0.18 | 0.24 | 0.70 | |
| Continuing phase | |  | 0.05 | 0.05 | 0.24 | 0.70 | |
| Terminal phase, non-CRC death | | | 0.05 | 0.05 | 0.24 | 0.70 | |
| Terminal phase, CRC death | | | 0.70 | 0.70 | 0.70 | 0.70 | |

# References

1. Hanmer J, Lawrence WF, Anderson JP, Kaplan RM, Fryback DG. Report of nationally representative values for the noninstitutionalized US adult population for 7 health-related quality-of-life scores. Med Decis Making. 2006;26(4):391-400. Epub 2006/07/21. doi: 26/4/391 [pii] 10.1177/0272989X06290497. PubMed PMID: 16855127

2. Swan JS, Kong CY, Hur C, Halpern EF, Itauma O, Williams O, et al. Comparing morbidities of testing with a new index: screening colonoscopy versus core-needle breast biopsy. J Am Coll Radiol. 2015;12(3):295-301. doi: 10.1016/j.jacr.2014.08.014. PubMed PMID: 25441485.

3. Jonas DE, Russell LB, Sandler RS, Chou J, Pignone M. Patient time requirements for screening colonoscopy. Am J Gastroenterol. 2007;102(11):2401-10. PubMed PMID: 17608779.

4. Ness RM, Holmes AM, Klein R, Dittus R. Utility valuations for outcome states of colorectal cancer. Am J Gastroenterol. 1999;94(6):1650-7. Epub 1999/06/11. doi: S0002-9270(99)00213-0 [pii]

10.1111/j.1572-0241.1999.01157.x. PubMed PMID: 10364039.
