# Supplementary material for: Cost-effectiveness of a multitarget stool DNA test for colorectal cancer screening of Medicare beneficiaries
Source: PLoS One. 2019 Sep 4;14(9):e0220234. doi: 10.1371/journal.pone.0220234 (PMC6726189; doi:10.1371/journal.pone.0220234)
Supplement: S4 Table — — = default strategy (i.e., the least costly and least effective non-dominated strategy); COL = colonoscopy; CRC = colorectal cancer; D = dominated; FIT = fecal immunochemical test; gFOBT = high sensitivity guaiac-based fecal occult blood test; ICER = incremental cost-effectiveness ratio; LYG = life-years gained compared with no screening; mtSDNA = multitarget stool DNA test; SIG = flexible sigmoidoscopy. * Future costs and life-years are discounted at a 3% annual rate. † Indicates a dominated strategy is weakly dominated (i.e., one of the other strategies provides more life-years gained than this strategy, and it has a lower incremental cost-effectiveness ratio). All other dominated strategies are strongly dominated (i.e., provide fewer life-years gained and have higher total costs than another strategy). (DOCX) [file pone.0220234.s007.docx]

|  | **CRC-SPIN** | | | | |  | **MISCAN** | | | | |  | **SimCRC** | | | | |
| --- | --- | --- | --- | --- | --- | --- | --- | --- | --- | --- | --- | --- | --- | --- | --- | --- | --- |
| **Strategy** | **CRC cases** | **CRC deaths** | **Lifetime costs,***  **million $** | **LYG*** | **ICER,**  **$** |  | **CRC cases** | **CRC deaths** | **Lifetime costs,***  **million $** | **LYG*** | **ICER,**  **$** |  | **CRC cases** | **CRC deaths** | **Lifetime costs,***  **million $** | **LYG*** | **ICER,**  **$** |
| No screening | 72 | 27 | 3.507 | 0.0 | D |  | 67 | 28 | 3.438 | 0.0 | D |  | 70 | 28 | 3.586 | 0.0 | D |
| gFOBT 1y | 17 | 4 | 2.045 | 107.2 | -- |  | 33 | 7 | 3.059 | 99.6 | D |  | 20 | 4 | 2.394 | 113.7 | -- |
| FIT 1y | 18 | 5 | 2.149 | 106.3 | D |  | 34 | 7 | 3.046 | 100.0 | -- |  | 21 | 4 | 2.402 | 113.7 | D |
| SIG 5y | 28 | 10 | 3.279 | 78.2 | D |  | 28 | 7 | 3.811 | 94.5 | D |  | 21 | 7 | 3.288 | 97.9 | D |
| SIG 10y + gFOBT 1y | 12 | 3 | 2.532 | 112.1 | D† |  | 27 | 6 | 3.528 | 105.9 | D† |  | 14 | 3 | 2.850 | 118.1 | 104,100 |
| SIG 10y +  FIT 1y | 13 | 3 | 2.641 | 111.6 | D |  | 27 | 6 | 3.591 | 106.8 | 80,700 |  | 15 | 3 | 2.900 | 118.1 | D† |
| COL 10y | 7 | 2 | 2.667 | 117.5 | 60,300 |  | 24 | 5 | 3.737 | 106.9 | 1,007,200 |  | 11 | 3 | 3.039 | 119.8 | 108,000 |
| mtSDNA 3y | 21 | 6 | 4.385 | 97.8 | D |  | 37 | 8 | 5.241 | 93.1 | D |  | 24 | 5 | 4.685 | 109.0 | D |
