# Supplementary material for: Cost-effectiveness of a multitarget stool DNA test for colorectal cancer screening of Medicare beneficiaries
Source: PLoS One. 2019 Sep 4;14(9):e0220234. doi: 10.1371/journal.pone.0220234 (PMC6726189; doi:10.1371/journal.pone.0220234)
Supplement: S5 Table — — = default strategy (i.e., the least costly and least effective non-dominated strategy); COL = colonoscopy; CRC = colorectal cancer; D = dominated; FIT = fecal immunochemical test; gFOBT = high sensitivity guaiac-based fecal occult blood test; ICER = incremental cost-effectiveness ratio; QALYG = quality-adjusted life-years gained compared with no screening; mtSDNA = multitarget stool DNA test; SIG = flexible sigmoidoscopy. * Future costs and quality-adjusted life-years are discounted at a 3% annual rate. † Indicates a dominated strategy is weakly dominated (i.e., one of the other strategies provides more quality-adjusted life-years gained than this strategy, and it has a lower incremental cost-effectiveness ratio). All other dominated strategies are strongly dominated (i.e., provide fewer quality-adjusted life-years gained and have higher total costs than another strategy). (DOCX) [file pone.0220234.s008.docx]

|  | **CRC-SPIN** | | |  | **MISCAN** | | |  | **SimCRC** | | |
| --- | --- | --- | --- | --- | --- | --- | --- | --- | --- | --- | --- |
| **Strategy** | **Lifetime costs,***  **million $** | **QALYG*** | **ICER,**  **$** |  | **Lifetime costs,***  **million $** | **QALYG*** | **ICER,**  **$** |  | **Lifetime costs,***  **million $** | **QALYG*** | **ICER,**  **$** |
| No screening | 3.928 | 0 | D |  | 3.966 | 0 | D |  | 4.086 | 0 | D |
| gFOBT 1y | 2.387 | 85.2 | -- |  | 3.599 | 73.0 | D |  | 3.134 | 81.7 | D |
| FIT 1y | 2.485 | 83.7 | D |  | 3.561 | 73.7 | -- |  | 3.131 | 81.8 | -- |
| SIG 5y | 3.110 | 68.9 | D |  | 3.878 | 78.2 | D |  | 3.603 | 73.4 | D |
| SIG 10y + gFOBT 1y | 2.489 | 95.8 | D |  | 3.747 | 86.4 | 14,700 |  | 3.282 | 90.4 | 17,400 |
| SIG 10y +  FIT 1y | 2.581 | 95.2 | D |  | 3.782 | 86.6 | D† |  | 3.320 | 90.6 | D† |
| COL 10y | 2.479 | 105.0 | 4,700 |  | 3.846 | 89.6 | 30,900 |  | 3.406 | 94.8 | 28,700 |
| mtSDNA 3y | 3.887 | 75.4 | D |  | 4.889 | 68.7 | D |  | 4.512 | 77.8 | D |
