# Supplementary material for: Cost-effectiveness of a multitarget stool DNA test for colorectal cancer screening of Medicare beneficiaries
Source: PLoS One. 2019 Sep 4;14(9):e0220234. doi: 10.1371/journal.pone.0220234 (PMC6726189; doi:10.1371/journal.pone.0220234)
Supplement: S1 Appendix — (DOCX) [file pone.0220234.s009.docx]

# Calculation of screening costs

Since the implementation of the Patient Protection and Affordable Care Act [[1](#_ENREF_1)], reimbursement rules have changed. As of January 2011, if a procedure was performed for screening and no biopsies or polypectomies were performed, a screening code (i.e., no biopsy or polypectomy) is used and patient coinsurance is waived (i.e., CMS reimburses 100% of the cost). If the procedure is diagnostic (i.e., follow-up exam after a positive stool-based test, regardless of findings) or therapeutic (i.e., biopsy or polypectomy is performed, regardless of whether it is a follow-up exam or originally for screening purposes), CMS covers 80% of the cost and the beneficiary is responsible for the remaining 20%. Because our analysis is from the perspective of CMS, these reimbursement rules are factored into all of our cost estimates.

Payments for the stool-based tests (i.e., gFOBT, FIT, and mtSDNA) were based on the 2017 Clinical Laboratory Fee Schedule [[2](#_ENREF_2)]. Average payments for endoscopic procedures (i.e., sigmoidoscopy and colonoscopy) were calculated from data provided by CMS and were based on 2014 outpatient Medicare claims data from the Chronic Conditions Data Warehouse (CCW).[[3](#_ENREF_3)] For this analysis, three places of service were considered: physician office setting, outpatient prospective payment system (OPPS), and ambulatory surgical center (ASC). We excluded claims for inpatient endoscopic procedures because screening, follow-up, and surveillance endoscopies are not typically performed in that setting. For procedures performed in the OPPS or ASC setting, we included associated facility charges. Payments were updated to 2017 dollars using the Personal Health Care Deflator price index [[4](#_ENREF_4)] (**S1 Table**). Estimated costs also include those associated with anesthesia services and pathology when provided in conjunction with an endoscopy.

If multiple lesions are detected within a single colonoscopy and all are biopsied or removed using the same technique, payment for the colonoscopy remains the same as it would be if only one lesion was intervened upon. However, if different types of polypectomy are required for the removal of multiple polyps, CMS reimburses 100% for the most expensive procedure code, and for each additional procedure code it reimburses the difference between that procedure and the base endoscopy code (i.e., the code for basic washing of the colon (CPT 45378)) [[5](#_ENREF_5)]. These reimbursement rules were captured in the data from the CCW provided by CMS that reported total payments by CMS for unique colonoscopies in which one or more surgical codes were submitted (i.e., CPT codes 45380-45381, 45383-45385. See **S2 Table** for code descriptions). Estimates of the average cost per screening test based on these assumptions are provided in **Table 2**.

## References

1. Patient Protection and Affordable Care Act, Pub. L. No. 111–148, 42 U.S.C. §§ 18001-18121, (2010).

2. Centers for Medicare and Medicaid Services. 2017 Clinical Laboratory Fee Schedule (CLAB) Public Use File (PUF). Available at <https://www.cms.gov/Medicare/Medicare-Fee-for-Service-Payment/ClinicalLabFeeSched/Clinical-Laboratory-Fee-Schedule-Files-Items/17CLAB.html>. 2017.

3. Chronic Condition Data Warehouse [cited 2016 January 13, 2016]. Available from: [www.ccwdata.org](file:///\\storage.erasmusmc.nl\v\vcl13\MAGE\DATA\UserData\594009\Colon\StoolDNA\Submissions\PlosOne\Revisions_2ndRound\Final\www.ccwdata.org).

4. <https://www.cms.gov/Research-Statistics-Data-and-Systems/Statistics-Trends-and-Reports/NationalHealthExpendData/Downloads/Tables.zip>,

“Table 23. National Health Expenditures; Nominal Dollars, Real Dollars, Price Indexes, and Annual Percent Change: Selected Calendar Years 1980-2017”

(row 41). .

5. Centers for Medicare and Medicaid Services. MLN Matters: Payment for Multiple Surgeries in a Method II Critical Access Hospital (CAH) 2012. Available from: [www.cms.gov/Outreach-and-Education/Medicare-Learning-Network-MLN/MLNMattersArticles/downloads/MM7587.pdf](file:///\\storage.erasmusmc.nl\v\vcl13\MAGE\DATA\UserData\594009\Colon\StoolDNA\Submissions\PlosOne\Revisions_2ndRound\Final\www.cms.gov\Outreach-and-Education\Medicare-Learning-Network-MLN\MLNMattersArticles\downloads\MM7587.pdf)
